# Is It Possible to Intervene in the Capacity of *Trypanosoma cruzi* to Elicit and Evade the Complement System?

**DOI:** 10.3389/fimmu.2021.789145

**Published:** 2021-12-16

**Authors:** Galia Ramírez-Toloza, Lorena Aguilar-Guzmán, Carolina Valck, Smrithi S. Menon, Viviana P. Ferreira, Arturo Ferreira

**Affiliations:** ^1^ Department of Preventive Veterinary Medicine, Faculty of Veterinary Medicine and Livestock Sciences, University of Chile, Santiago, Chile; ^2^ Department of Pathology, Faculty of Veterinary Medicine and Livestock Sciences, University of Chile, Santiago, Chile; ^3^ Department of Immunology, Institute of Biomedical Sciences (ICBM), Faculty of Medicine, University of Chile, Santiago, Chile; ^4^ Department of Medical Microbiology and Immunology, College of Medicine and Life Sciences, University of Toledo, Toledo, OH, United States

**Keywords:** *Trypanosoma cruzi*, host-parasite interaction, complement system, complement regulatory proteins, host-immune evasion

## Abstract

Chagas’ disease is a zoonotic parasitic ailment now affecting more than 6 million people, mainly in Latin America. Its agent, the protozoan *Trypanosoma cruzi*, is primarily transmitted by endemic hematophagous triatomine insects. Transplacental transmission is also important and a main source for the emerging global expansion of this disease. In the host, the parasite undergoes intra (amastigotes) and extracellular infective (trypomastigotes) stages, both eliciting complex immune responses that, in about 70% of the cases, culminate in permanent immunity, concomitant with the asymptomatic presence of the parasite. The remaining 30% of those infected individuals will develop a syndrome, with variable pathological effects on the circulatory, nervous, and digestive systems. Herein, we review an important number of *T. cruzi* molecules, mainly located on its surface, that have been characterized as immunogenic and protective in various experimental setups. We also discuss a variety of parasite strategies to evade the complement system - mediated immune responses. Within this context, we also discuss the capacity of the *T. cruzi* infective trypomastigote to translocate the ER-resident chaperone calreticulin to its surface as a key evasive strategy. Herein, it is described that *T. cruzi* calreticulin inhibits the initial stages of activation of the host complement system, with obvious benefits for the parasite. Finally, we speculate on the possibility to experimentally intervene in the interaction of calreticulin and other *T. cruzi* molecules that interact with the complement system; thus resulting in significant inhibition of *T. cruzi* infectivity.

## Introduction

Chagas disease, or American trypanosomiasis, is a multisystemic disorder that affects the cardiovascular, digestive, and central nervous systems (1). Chagas disease is one of the 20 most “neglected tropical diseases”, as defined by The World Health Organization (WHO) (2). About 6-7 million people are infected worldwide, with almost 100 million at risk, indicating that this disease is a serious public health issue (3, 4). In endemic countries, Chagas disease is primarily transmitted by triatomine vectors, predominantly in rural areas. However, human migration and other forms of transmission have changed the epidemiology/epizootiology of Chagas disease, which is currently affecting peri-urban and urban areas (5, 6). Other important mechanisms of transmission include blood transfusion, organ transplants, oral ingestion, laboratory accidents, vertical transmission from mother to child, or needle sharing (2, 7).

Chagas disease is caused by *Trypanosoma cruzi* (*T. cruzi*), a hemoflagellate parasite transmitted through various species of hematophagous reduviid insects (‘kissing bugs’) mainly in endemic areas such as Latin America (8). Trypomastigotes, the infective form, circulate in the blood of mammals and infect nucleated cells, where they transform into amastigotes, the replicative form. *T. cruzi* needs to evade the host immune system, especially during the acute phase of the infection, and various mechanisms have been described for the parasite to control the innate and adaptive host immune responses. In the regulation of adaptive immune responses, inhibition of polyclonal activation of B and T cells may be relevant in infected people (9) and in mice (10, 11). Additionally, a decrease in the proliferative response of lymphocytes, as well as in the production of interleukin-2 (IL-2) in chronic Chagas disease patients has also been reported (12). Moreover, the parasites induce immunomodulatory molecules, such as IL-10 and transforming growth factor-β (TGF-β), which lead to failure in the maturation of antigen-presenting cells and poor antigenic presentation (12).

**Figure 1:**
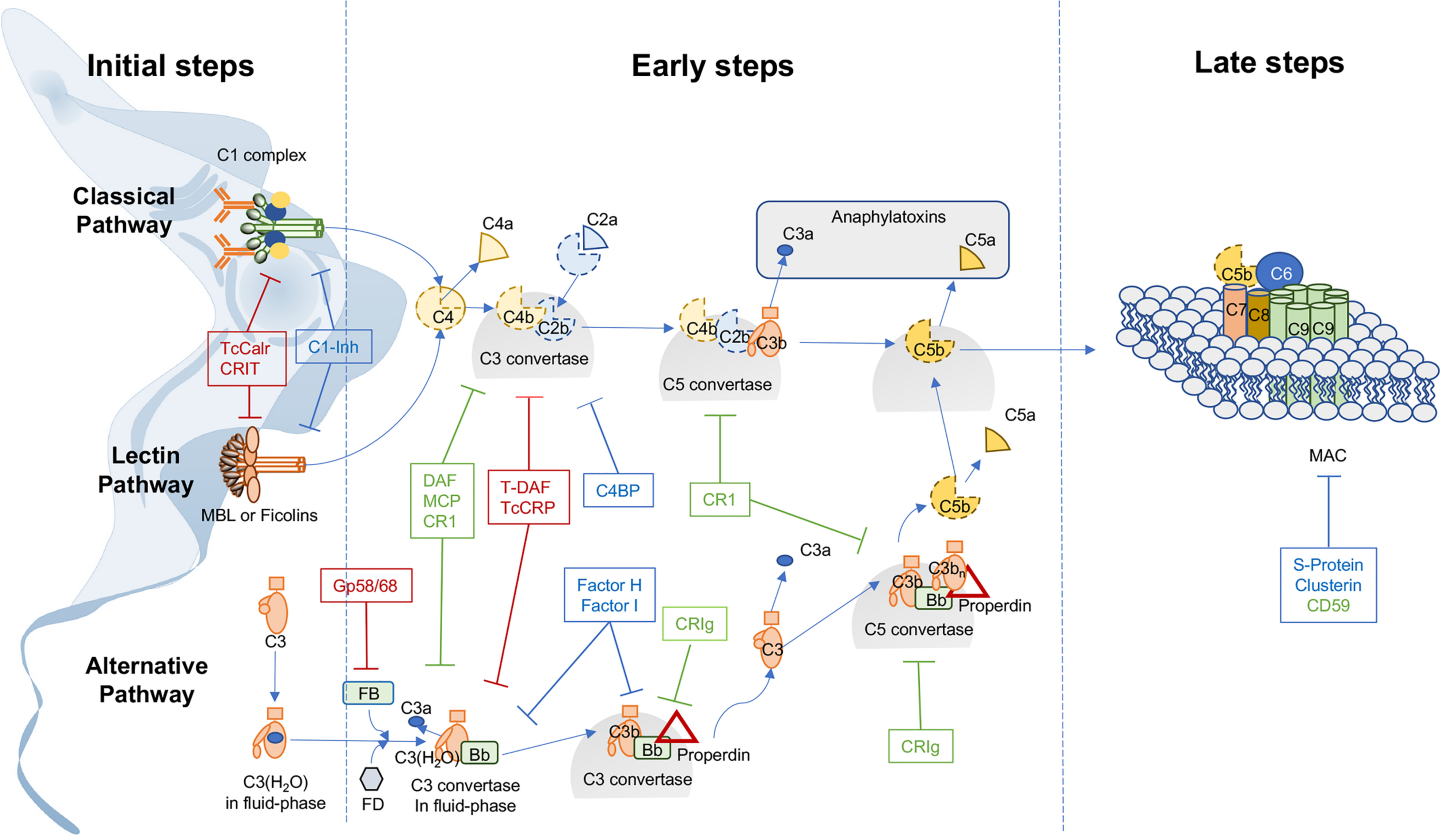
*Trypanosoma cruzi* expresses, secretes, or recruits complement regulatory proteins and intervening in the interaction of these *T. cruzi*-derived regulatory proteins with complement can affect host-parasite interactions. The complement system (C) is activated by three different pathways: classical (CP), lectin (LP) and alternative (AP). (1) In the initiation steps, these pathways are activated by the identification of different pathogen-associated molecular patterns (PAMPs) present on microorganisms such as *T. cruzi*. Thus, the CP is activated when C1 complex (C1qr_2_s_2_) recognizes antibodies bound to *T. cruzi* or acute phase proteins. The LP is activated when MBL and ficolins form complexes with serine proteases (MASPs) in the presence of carbohydrates. The AP is activated by spontaneous hydrolysis of C3, near a variety of non-self cell surfaces. (2) In the early steps, all activated pathways converge in the generation of C3 convertases, that continuously cleave C3 into C3a and C3b continuing with the enzymatic cascade that also generates C5 convertases that produce the split products C5a and C5b. (3) Finally, in the late step, C5b anchored to the pathogen surfaces, in conjunction with C6-C9, form the membrane attack complex (MAC) and lyse the pathogen. Thus, C activation induces opsonization (by C3b and C4b), inflammation (by C3a and C5a) and lysis of microorganisms such as *T. cruzi*. However, C activation is stringently controlled by C regulatory proteins. The membrane bound regulatory proteins are: Decay-accelerating factor (DAF), membrane co-factor protein (MCP), C receptor 1 (CR-1) CD59 and complement receptor of immunoglobulin family (CRIg) (in green). Regulatory proteins found in plasma are: Factor I, Factor H, C4 binding protein (C4BP), C1-inhibitor (C1-Inh), S-protein and Clusterin (in blue). These proteins limit amplification of the downstream cascade. To evade C activation, *Trypanosoma cruzi* expresses and secretes complement regulatory proteins with homologous function with their human counterparts (in red). Thus, CRIT and TcCalr inhibit C in early stages of activation, and T-DAF, TcCRP and gp58/68 participate in intermediate stages of activation.

To evade the innate immune response, one of the most important mechanisms adopted by *T. cruzi* is to modulate complement system (C) activity (**Figure 1**). Thus, infective trypomastigotes, are resistant to C, while non-infective epimastigotes, present in the reduviid insect vector, are extremely sensitive (13, 14). However, this C resistance varies among *T. cruzi* strains (15), being mediated by (a) surface expression of molecules such as glycoprotein 58/68 (gp 58/68) (16), *T. cruzi* complement regulatory protein (TcCRP) (17–19), *T. cruzi* trypomastigote-decay accelerating factor (T-DAF) (20, 21), *T. cruzi* calreticulin (TcCalr) (22), C2 receptor inhibitor trispanning (CRIT) (**Table 1**) and/or (b) secretion or acquisition of molecules from host blood stream, such as Factor H (FH) (36), and *T. cruzi* induced host extracellular vesicles (EV) (37). These molecules inhibit C at the initial steps of the cascade or inhibit C3 and/or C5 convertases of the classical (CP), lectin (LP) and/or alternative (AP) pathways (37) (**Figure 1**). However, studies on their therapeutic or prophylactic values are still limited. Herein, we will focus on the interactions of these molecules with C, and in their potential therapeutic/prophylactic roles.

**Table 1:** Complement regulatory proteins expressed and/or secreted by *Trypanosoma cruzi*, their roles in the host-parasite interaction and as potential therapeutic or prophylactic tools.

Complement regulatory protein	Functions in Complement system evasion	Other roles in the host-parasite interaction	Therapeutic or prophylactic potential	Reference
*Trypanosoma cruzi* Complement C2 Receptor Inhibitor Trispanning Protein (CRIT)	CRIT is a 32 kDa protein that inhibits the C2 cleavage by C1s and MASP2 and impairs C3 convertase formation in CP and LP.	Undetermined	Undetermined	(23, 24)
*Trypanosoma cruzi* calreticulin (TcCalr)	TcCalr is a 45 kDa protein expressed on the parasite surface and secreted that inhibits the CP and LP in initial step of activation. TcCalr binds to C1, MBL and L-Ficolin.	TcCalr is highly immunogenic in humans and mice and binds C1q, promoting infectivity. Additionally, TcCalr inhibits angiogenesis and tumor growth.	Recombinant TcCalr and DNA-based immunization promote higher parasitemias. Anti - TcCalr F(ab’)_2_ antibody fragments reduce parasitemia and increase survival in mice.	(22, 25–29)
Trypomastigote Decay-Accelerating Factor (T-DAF)	T-DAF is an 87-93 kDa glycoprotein expressed on the parasite surface that interferes with assembly of the C3 and C5 convertase of both CP, LP (probably) and AP.	Highly immunogenic in humans and mice.	Recombinant T-DAF immunization promotes antibody production in different animal species, leading to parasite lysis *in vitro*.	(20, 21, 30)
*Trypanosoma cruzi* Complement Regulatory Protein (TcCRP)	TcCRP is a glycoprotein, also named gp160, expressed on the parasite surface that binds C3b and C4b, inhibiting the CP and AP C3 convertase. TcCRP inhibits the CP, LP (probably) and AP.	TcCRP is highly immunogenic and induces lytic antibodies in humans and mice.	TcCRP DNA-based immunization protects against *T. cruzi* infection in mice.	(17–19, 31–34)
Glycoprotein 58/68 (Gp58/68)	Gp58/68 is a 58-68 kDa protein expressed on the parasite surface that interferes with the C3 convertase formation by binding Factor B, thus specifically inhibiting the AP.	Gp58/68, first described as a receptor to fibronectin, has a likely role in infectivity.	Undetermined	(16, 35)

CP, Classical pathway; LP, Lectin pathway; AP, Alternative pathway; C, Complement system.

## *T. cruzi* Molecules Inhibiting C at the Initial Steps

*T. cruzi* complement C2 receptor inhibitor trispanning protein (CRIT), a 32 kDa protein containing a 27 amino acid extracellular domain (38–40), is a C2 receptor, present on *T. cruzi*, that inhibits C2 cleavage by C1s (38). First described in the Y strain, CRIT expressed on trypomastigotes binds to, and inactivates C2, inhibiting the CP and LP (23). The same group then showed that in the LP, the extracellular domain 1 of CRIT inhibits MBL-Associated Serine Protease-2 (MASP-2) mediated C2 cleavage, thus impairing formation of the C3 convertase (24). Thus, parasites overexpressing CRIT are highly resistant to C-mediated lysis (24). CRIT is expressed in different *T. cruzi* strains and clones, such as CL Brenner, Colombiana and Dm28c, with high sequence identity (88 - 98%) (23), but apparently its gene is only functional in some *T. cruzi* lineages. A recent study evaluating the resistance of C in different TcI strains with high (Qro) and low (Ninoa) virulence, demonstrated that the mRNA of CRIT is three – fold lower in the low virulence strain (41).

*T. cruzi* calreticulin (TcCalr) (formerly known as TcCRT), is a highly pleiotropic protein, with inhibitory effects in C activation and infectivity. In addition to these roles, TcCalr, also reduces angiogenesis and tumor growth, however, these roles have been described and reviewed elsewhere (25, 42–46). Infective trypomastigotes carrying a monoallelic deletion of the *TcCalr* gene, are significantly susceptible to C-mediated lysis. On the contrary, parasites overexpressing TcCalr are significantly more resistant to CP and LP-mediated lysis (26, 47). TcCalr binds to the collagenous tails of C1q, inhibiting the CP (22). Its central TcCalr S-domain (aa 159-281) competes with the (C1r-C1s)_2_ tetrameric complex to bind C1q, thus decreasing C4b generation and in turn decreasing the levels of the generated CP C3 and C5 convertases (22). Furthermore, both CP serine-proteases, C1s and C1r, bind TcCalr ***in vitro***, but TcCalr does not inhibit the C4-activating function of solid phase-bound C1s. Perhaps, C1s inactivation occurs only when the serine protease is part of C1 complex (C1q, (C1r, C1s)_2_) (27). Additionally, TcCalr competes with the capacity of the serine proteases to bind C1q, but does not displace them from the preformed C1 complex (27). In Chagas disease, this role may be also important in other steps of the parasite cycle, since *Triatoma infestans* calreticulin (TiCalr), present in the insect’s saliva, also binds C1, inhibiting the CP. Perhaps, TiCalr prevents mammal C-mediated damage to the vector’s digestive mucosa (48). TcCalr and its S-domain (aa 159-281) also binds mannan-binding lectin (MBL) and Ficolins, inhibiting the LP (22, 28). Although TcCalr binds to the collagenous tails of MBL and reduces the binding of MBL to mannose, it does not inhibit C4 activation (22). On the other hand, L-Ficolin (but not H-Ficolin) binds to TcCalr, interfering with its activation ***via*** Lipoteichoic-acid. Moreover, because trypomastigotes translocate significantly higher amounts of TcCalr to their surfaces, L-Ficolin binds preferentially to this infectious stage of the parasite (28). Thus, TcCalr inhibits both CP and LP (22, 27, 28).

TcCalr is an ER-resident protein that translocates to the parasite external microenvironment. Although TcCalr is located mainly in the ER, it is also found in the Golgi, reservosomes, flagellar pocket, cell surface, cytosol, nucleus and kinetoplast (22, 49, 50). Thus, C1q and TcCalr colocalize on the parasite surface, mainly on the area of flagellar emergence (16). It is well known that CALR (the human TcCalr counterpart), participates as an *“eat me”* signal in apoptotic cancer cells, promoting their phagocytosis (51). This process is mediated by the CALR/C1q interaction on the apoptotic cells, which is recognized, in turn, by a C1q receptor (also identified as membrane bound CALR) on the phagocytic cell (52). Therefore, the TcCalr – C1q interaction underlies a molecular mimicry strategy to enhance parasite internalization. In agreement with these findings, tissue-culture trypomastigotes bind C1q, increasing internalization into monocytes and macrophages (51). However, recombinant TcCalr (rTcCalr) and DNA-based immunization induces specific antibody production and promotes higher parasitemias in mice (29). This apparent paradox is resolved when anti-TcCalr F(ab’)_2_ antibodies are used to inhibit the TcCalr/C1q interaction *in vivo*, demonstrating that Fc-antibody regions recruit C1q thus promoting higher infectivity (29, 53). Unlike the infective forms, epimastigotes are highly sensitive to C activation, most likely due, at least in part, to the marginal levels of TcCalr expressed on their surfaces (22, 44). However, when TcCalr is exogenously added to non-infective epimastigotes, the parasites are internalized by fibroblasts in a C1q-dependent manner (54). Additionally, mice inoculated with genetically modified trypomastigotes, under-expressing TcCalr, did not generate detectable parasitemia nor anti-*T. cruzi* IgG antibodies. Accordingly, parasites under-expressing TcCalr showed a reduced capacity to evade the C and to infect cells (26).

TcCalr-C1q interaction is also relevant in human placenta which expresses high CALR levels (55–57). In an *ex vivo* model, TcCalr is shown to bind C1q (58) and possibly recognized by cC1qR (a membrane-bound CARL form) present on the placental syncytiotrophoblast. This interaction is also inhibited by polyclonal F(ab’)_2_ anti-TcCalr antibodies, a fact reflected in lower parasite infectivity in an *ex vivo* experimental model. An *in vivo* infectivity inhibitory capacity for anti-TcCalr antibody fragments can be envisaged, considering that, in humans, congenital transmission ranges from 2% to 13.8% in different studies (59).

As mentioned, TcCalr also binds to MBL and L-Ficolin (22, 27). However, the potential role of TcCalr-MBL or TcCalr-Ficolin interactions, in the infectivity process, requires additional research. One study comparing two *T. cruzi* strains, susceptible and resistant to C, suggested that MBL also participates in the infectivity process while the parasite deactivates the LP (60). Nevertheless, the complete inactivation of the LP does not confer higher susceptibility to the infection since, mice deficient in MASP-2 show similar parasitemia and survival compared to wild-type (61). This fact may indicate that the LP is not essential to control parasitemia and infectivity. However, low levels of L-Ficolin and *FCN2* (gene codifying for L-Ficolin) polymorphism are associated with chronic Chagas disease (62).

Based on *in silico* structural TcCalr models, an interesting peptide (VC-TcCalr), at the TcCalr N-domain, has been delimited and chemically synthesized. VC-TcCalr is a strong dipole, spatially stable (more than its CALR counterpart), that interacts with collagen-like tails and scavenger receptors. This peptide binds to C1q and was anti-angiogenic in a *Gallus gallus* chorioallantoic membrane assays (63). This crystallographic structural study defines CALR conformational rearrangements that could be informative in future therapeutic investigations of parasite CALR (64), mainly in its anti-complement and anti-neoplastic effects.

## *T. cruzi* Molecules That Inhibit C3 and C5 Convertases

Metacyclic, bloodstream and tissue culture-derived *T. cruzi* trypomastigotes express an 87-93 kDa glycoprotein (T-DAF), with decay accelerating activity on the CP and AP C3 and C5 convertases (20, 21). This activity was previously found in human decay-accelerating factor (DAF), a 70 kDa glycophospholipid-anchored membrane protein. DAF is present on erythrocytes, neutrophils, lymphocytes, monocytes, platelets, and endothelial cells (65). T-DAF is functionally, but not structurally analogous to human DAF (21). T-DAF mRNA levels are lower in C-susceptible *T. cruzi* strains (41). A partial T-DAF cDNA clone and its deduced protein sequence showed 40% homology with a portion of the coding region for DAF (21). T-DAF is immunogenic in experimental animals, inducing antibodies with parasitic lysis capacity (21). Additionally, antibodies against T-DAF were identified in patients chronically infected with *T. cruzi* (21, 66, 67); thus, T-DAF is highly immunogenic in both humans and mice, suggesting a serodiagnosis value (30).

*T. cruzi* C regulatory protein (TcCRP), also named gp160, is a 160 kDa GPI-anchored glycoprotein (17) that can be spontaneously released by the trypomastigotes. This protein can inhibit both the CP and AP and stable TcCRP-transfected epimastigotes were found to be protected from C-mediated lysis (31). There are multiple copies of TcCRPs in the *T. cruzi* genome, highlighting the importance of this protein for *T. cruzi*. The encoded proteins are not only structurally and functionally similar to DAF (17, 18, 31), but are also similar to members of the *T. cruzi*-Trans-Sialidase (TS) superfamily (68). Proteins from this superfamily have enzymatic capacity to transfer monosaccharides from host sialyl-glycoconjugates to terminal β-galactoses of acceptor molecules located on the parasite surface, thus contributing to the parasite survival. However, this superfamily is classified in eight groups, where only group-I has enzymatic activity, and groups II-VIII are considered inactive (68). TcCRP promotes evasion of immune response, in a TS-independent manner (69–71). As expected, positive correlations between the virulence of *T. cruzi* strains and TcCRP expression levels (72) or mRNA levels (41) have been described. Moreover, TcCRP is immunogenic and induces lytic antibodies in humans and mice (32) and a DNA-based immunization confers protection against *T. cruzi* infection in mice (33). The levels of lytic antibodies induced by TcCRP in mice infected with different *T. cruzi* strains suggested that higher levels of parasitemia resulted in an increased exposition of TcCRP and other proteins, which bind to lytic antibodies present in the host’s blood (34). Thus, humoral immune responses, including lytic antibody secretion, could play a role in the later replication cycle, promoting phagocytosis and cellular cytotoxicity to control the infection (34). Additionally, TcCRP is phylogenetically similar to FL-160, a TS-like protein located in the *T. cruzi* flagellum and flagellar pocket, with still unexplored functions (73). FL-160 derived peptides, presented by the MHC class I pathway (74) (recognized by CD8+ T cells), may have a pathogenic or protective role in chronic Chagas disease (75).

Trypomastigote glycoprotein 58/68 (gp 58/68) (58 or 68 kDa, under non-reducing or reducing conditions, respectively) (35) also inhibits C. In cell-bound and fluid-phase conditions, the protein is shown to have a dose-dependent decay-accelerating activity on the AP C3 convertase formation. However, it does not enhance the decay-dissociation of preformed AP C3 convertases and does not serve as a co-factor for Factor I (FI). Therefore, its inhibitory effect may depend on its interaction with Factor B rather than with C3b (16).

## Other Molecules and Mechanisms Related to *T. cruzi* C Evasion

Factor H (FH), a 155 kDa fluid-phase C negative regulatory protein, composed of 20 short consensus repeats (SCR) (76, 77), can accelerate the decay of the surface-bound AP C3 and C5-convertases (78). In *T. cruzi*, FH binds with higher affinity to C3b bound to metacyclic trypomastigotes than to epimastigotes (36). FH uses 3 specific sites (79, 80) to interact with unique domains on C3b (79, 81–83), participates as a cofactor for Factor I (FI) and interacts with sialic acid and other related molecules (78, 84, 85). This property is important for *T. cruzi*, because the parasite has TS to transfer the polyanion α (2, 3)-linked sialic acid from serum glycoconjugates to acceptor sites on the parasite surface (86, 87). These glycoconjugates contribute to regulate C activation on the parasite surface, thus behaving as a virulence factor (88–91). As sialylated molecules downregulate AP activation (92, 93), these polyanions transferred by TS on the trypomastigote surface may also be critical for survival in the circulation (94, 95). This is supported by other studies that highlight the relationship between FH and sialic acid to control C activity in parasites such as *Toxoplasma gondii* (96), *Plasmodium falciparum* (97–99) and *Echinococcus granulosus* cysts (100). Moreover, a positive correlation has been described between FH plasma level and inflammation, cardiac involvement and cardiometabolic parameters in chronic Chagas disease (101).

Extracellular vesicles (EVs) are described in several infectious- and non-infectious diseases and stress (102–107). Bloodstream and endothelial cells release EVs from their cellular membranes (107–110). EVs participate in intercellular communication, transferring glycoproteins, lipids, nucleic acids, and other biomolecular cargos. EVs may play an important role in the parasite-host cell dynamics and in the physiopathology of Chagas disease (111). *T. cruzi* trypomastigotes are exposed to host cell EVs and also induce EVs release from blood cells in a Ca^2+^-dependent manner. These vesicles bind to C3 convertase, inhibiting the catalytic activity of both the CP and LP (37). On the other hand, EVs with a TGF-β cargo promote host cell invasion ***via*** the lysosome-independent route. This phenomenon is dose- and parasite- infective stage dependent and non-specific for parasite strains or host cell types (37). In agreement with this, higher levels of TGF-β are found circulating in chronically infected patients (112). Thus, TGF-β-bearing EVs could activate the TGF-β signaling pathway to promote parasite infectivity (37). Simultaneously, *T. cruzi* produces exosomes that stimulate different host cells to produce EVs and modulate the immune response (37, 113). EVs contribute to C-resistance and infectivity in trypomastigotes (114), but this response is strain-dependent since EVs derived from a more C-resistant strain can affect infectivity rate of another more susceptible *T. cruzi* strain (115). On the other hand, infected mice in the presence of *T. cruzi*-derived EVs present higher parasitemia (37, 116), and mice pre-inoculated with EVs, before infection, register higher mortality or severe pathology (117). The composition of EVs has been evaluated by proteomic and transcriptomic analysis. However, the size, amount and composition may vary according to strain, origin and life cycle stage, among others. Thus, EVs contain proteins related with metabolism, signaling, and virulence, some of them related with C evasion, such as TcCalr (115, 117–119).

## Future Therapeutic Perspective

Efforts to control Chagas disease have been mainly focused on programs aimed at the triatomine vectors. However, there is an urgent need to design new therapeutic and/or preventive tools since current treatments are not completely efficient and are seriously complicated by deleterious side effects (120). C regulatory proteins released by *T. cruzi* may represent therapeutic or immunogenic/antigenic targets. Given the importance of the C role in innate and adaptive immune response and that *T. cruzi* adopts various strategies to evade C, it is unfortunate that majority of *T. cruzi* C regulatory proteins have not been considered in vaccine designs or therapeutic strategies (120). Some advantages of considering C regulatory proteins expressed by *T. cruzi* as immunogens in vaccines are: 1) They intervene at different levels of the C cascade and several C routes, simultaneously; 2) Some of them participate in other mechanisms involved in the host-parasite interaction, such as infectivity; 3) Most of them are highly immunogenic and, 4) Despite sharing functions with host C regulatory proteins, they are not completely homologous to their human counterparts. However, since some of these proteins share mechanisms of action, inactivation of one molecule may cause inhibition at different levels or pathways of C activation. Therefore, the site of action of candidate molecules must be carefully experimentally dissected out. Another unexplored possibility is to consider targeting the interaction of C regulatory proteins that is being hijacked by *T. cruzi*. The specific inhibition of catalytic sites of proteins with enzymatic roles can be ascertained. This inhibition could be performed by antibodies, nanobodies, partially or completely humanized monoclonal antibodies or natural or synthetic competitor molecules.

## Authors Contributions

GR-T, VF, and AF contributed equally to the generation of this review. GR-T prepared the figure. GR-T, VF, and AF edited the text. LA-G, CV, and SM contributed substantially to the writing, researching previous published works, revision and approved the submitted version. All authors contributed to the article and approved the submitted version.

## Funding

The University of Toledo Biomedical Research Innovation Program (VF), Toledo, Ohio, USA; VID, University of Chile (AF); FONDECYT-Chile 1130099 (AF), CONICYT-REDES 170126 and FIV-FAVET 12101701-9102-181 (GR-T).

## Conflict of Interest

The authors declare that the research was conducted in the absence of any commercial or financial relationships that could be construed as a potential conflict of interest.

## Publisher’s Note

All claims expressed in this article are solely those of the authors and do not necessarily represent those of their affiliated organizations, or those of the publisher, the editors and the reviewers. Any product that may be evaluated in this article, or claim that may be made by its manufacturer, is not guaranteed or endorsed by the publisher.
